# A distance-based, randomized controlled trial for reducing sedentary behavior among prostate cancer survivors: a study protocol

**DOI:** 10.1186/s12889-022-13218-5

**Published:** 2022-04-28

**Authors:** Linda Trinh, Catherine M. Sabiston, Shabbir M. H. Alibhai, Jennifer M. Jones, Kelly P. Arbour-Nicitopoulos, Daniel Santa Mina, Kristin Campbell, Guy E. Faulkner

**Affiliations:** 1 Faculty of Kinesiology and Physical Education, University of Toronto, 55 Harbord Street, Toronto, Ontario M5S 2W6 Canada; 2 Faculty of Medicine, University of Toronto, 1 King’s College Circle, Medical Sciences Building, Toronto, ON M5S 1A8 Canada; 3 Toronto General Research Institute, Toronto General Hospital, 200 Elizabeth St, Toronto, ON M5G 2C4 Canada; 4 Department of Supportive Care, Princess Margaret Cancer Centre, 610 University Ave, Toronto, ON M5G 2C1 Canada; 5 Department of Physical Therapy, University of British Columbia, 2177 Wesbrook Mall, Vancouver, BC V6T 1Z3 Canada; 6 School of Kinesiology, University of British Columbia, 210-6081 University Boulevard, Vancouver, BC V6T 1Z1 Canada

**Keywords:** Prostate cancer, Sedentary behavior, Physical activity, Randomized controlled trial, Distance-based

## Abstract

**Background:**

Prostate cancer survivors (PCS) experience long-term side effects beyond treatment such as fatigue, depression and anxiety. Quality and engaging supportive care programs are needed to reduce these chronic and debilitating effects. Independent of physical activity (PA), high volumes of sedentary behavior (SB) are associated with chronic disease-related risk factors and poorer cancer-specific quality of life (QoL). Simultaneously increasing PA and decreasing SB may be an effective health promotion strategy. Given that PCS may face several barriers to engaging in supervised programs, there is a need to develop and assess the efficacy of interventions that employ distance-based approaches for behavior change. The primary aim of this study is to determine the effects of a 12-week intervention (Fitbit + behavioral counselling) vs. Fitbit-only control group in reducing SB among PCS. Secondary outcomes include light-intensity PA, QoL, motivational outcomes, and patient satisfaction.

**Methods:**

This two-armed, randomized controlled trial will recruit inactive PCS (stage I-IV) across Canada who self-report engaging in >8 hours/day of SB. Participants will be randomized to the intervention (*n*=60; Fitbit and behavioral support) or active control group (*n*=60; Fitbit-only). The intervention consists of the use of a Fitbit and a series of six behavioral support sessions (two group, four individual) to aid PCS in gradually replacing SB with light-intensity PA by increasing their daily step counts to 3,000 steps above their baseline values. The Fitbit-only control condition will receive a Fitbit and public health PA resources. The primary outcome is change in SB measured objectively using activPAL inclinometers. All secondary outcomes will be measured via self-report, except for PA which will be measuring using Fitbits. Data will be collected at baseline, post-intervention, and at 6-month post-intervention.

**Discussion:**

Reducing SB and increasing light-intensity PA plays an important, yet often undervalued role in the health and well-being of PCS. This study will create a unique distance-based platform that can be used by clinical and community-based organizations as a low-cost, supportive care tool to improve health outcomes for PCS.

**Trial Registration:**

ClinicalTrials.gov Identifier NCT05214937. Registered January 28, 2022

Protocol version: v.1

## Background

The ten-year relative survival rate for prostate cancer for all stages combined is 90%, resulting in a growing number of prostate cancer survivors (PCS) [1]. Many PCS suffer from long-term side effects well beyond treatment, such as urinary incontinence, erectile dysfunction, fatigue, depression, and anxiety [2–4]. Supportive care interventions are needed to reduce the chronic effects of cancer and its treatment during the transition into survivorship. Physical activity (PA) has a positive impact on many clinical outcomes, including improved quality of life (QoL), cancer-specific mortality, and reducing treatment-related toxicities among PCS [5–8]. Despite this, few PCS achieve current PA guidelines [9–11], and there is a significant decrease in PA during and after adjuvant therapy [12]. Short-term supervised PA programs have been successful in improving fitness and patient-reported outcomes in PCS [13], but uptake and adherence remain low [14].

Independent of PA, addressing prolonged periods of sedentary behavior (SB) has numerous beneficial health outcomes, including a reduced risk of mortality [15–19] and improved QoL [20–22]. SB is defined as any waking behavior characterized by a low energy expenditure (i.e., ≤1.5 resting metabolic equivalents) while in a sitting, reclining, or lying down posture [23]. In Canada, 5.8% of all associated cancers is attributable to SB, with increased cancer risk for ≥6 hours per day of SB [24]. PCS spend most of their day sedentary (i.e., 69% of waking hours) or engaged in light-intensity PA (LPA; 30% of waking hours) compared to moderate-to-vigorous intensity PA (MVPA; 1% of waking hours) [11], thus providing more opportunities to address SB in a day. As such, focusing on reducing SB among PCS may be a more feasible intervention approach than supervised exercise. A pooled analysis examining associations between PA, SB, and percentiles of QoL showed that ≥30-minute bouts of SB are inversely associated with functional well-being [20]. Similarly, LPA appeared to be beneficial for QoL even amongst those with the poorest QoL distributions [20]. Simultaneously increasing MVPA and reducing SB produced clinically relevant changes in QoL (i.e., physical and role function) for PCS [20, 25]. Despite the growing evidence that reduced SB may result in better QoL, there are no known effective strategies aimed at reducing SB among PCS. However, given the independent effects of increasing PA and minimizing time spent in SB on health outcomes, interventions should not only target reductions in sedentary time, but also replacing that behavior with light-intensity PA.

Developing theory-driven interventions is critical for facilitating adoption and maintenance of behavior change [26]. For successful behaviour change, it is necessary to move beyond intention-focused theories towards psychosocial and behavioural constructs proposed to reduce the intention-behaviour gap, which may be more effective for behavior change [26]. One such approach is the Multi-Process Action Control (M-PAC) framework, which builds on the well-established social cognitive antecedents of PA behavior, while also recognizing the intention-behavior gap [26]. The M-PAC framework has a layered, progressive structure where an individual moves from intention formation to adoption of action control and onto maintenance of action control. Intention formation is predicated on initiating reflective processes (i.e., instrumental attitude and perceived capability). Reflective processes are hypothesized to influence intention formation and initiate regulatory processes to enact this intention (i.e., affective attitude and perceived opportunity). The translation of intention into PA (i.e., action control) is determined partially by regulatory processes (e.g., action planning, coping planning, self-monitoring, social support) during the initial adoption of the behavior. Continuation of PA action control is thought to include the addition of reflexive processes (e.g., habit) for maintenance of behavior change. The utility of the M-PAC framework for successful behavior change has shown promise in an exercise telephone counselling intervention in hematological cancer survivors [27], however, no study to date has examined a theory-based intervention in SB in PCS or any other cancer survivor group.

Though supervised interventions have been effective in increasing PA behaviors among cancer survivors [13], distance-based interventions are an attractive alternative due to the ability to reach a large number of cancer survivors, accessibility, and lower cost. Wearable technology activity monitors (e.g., Fitbit) are a relatively low-cost, self-management tool that have a wide reach and broad applications for use in clinical and public health settings. Initial interventions using wearable technology activity monitors have shown promising improvements in PA and SB [28, 29]. A previous pilot study conducted by our research group examined the feasibility of a *m*health application (RiseTx) for reducing SB and increasing MVPA among PCS undergoing androgen deprivation therapy (ADT) [30]. PCS were given an activity tracker, access to the RiseTx *m*health web application, and a goal of increasing step counts by 3,000 daily steps above baseline levels over 12-weeks. A range of behavior change support tools were progressively deployed to reduce SB (e.g., self-monitoring of steps; action planning). The RiseTx *m*health application was successful in reducing device-measured SB by 455 minutes/week and increasing MVPA by 44 minutes/week, as well as increasing daily steps by 1535 between baseline and post-intervention [30]. The current study will build on this successful pilot study and harness the many lessons learned during the implementation of RiseTx. As an extension of this prior work, the current intervention will provide additional synchronous behavioral support aligned with processes in the M-PAC framework through videoconferencing to reduce SB. We are replacing the web-platform with 1:1 videoconferencing with a movement specialist in response to feedback from the original RiseTx participants.

The primary objective is to determine the effects of the 12-week intervention (i.e., Fitbit + behavioral counselling) compared to a control condition (Fitbit + public resources) in reducing SB in PCS. It is hypothesized that the intervention group will decrease their SB compared to the Fitbit-only control condition at post-intervention (12 weeks) and 6-month follow-up. Secondary objectives are to determine the effects of the intervention on secondary outcomes including changes in MVPA, LPA, motivational outcomes from the M-PAC framework, physical function and patient-reported outcomes (i.e., QoL, fatigue, disability and mental health) compared to the Fitbit-only control group.

## Methods/Design

This study protocol follows the SPIRIT guidelines [31]. Ethics approval was granted by the Research Ethics Board at the University of Toronto (protocol #41699). The trial was registered with the ClinicalTrials.gov database on January 28, 2022 (NCT05214937).

### Study Design

**Fig. 1:**
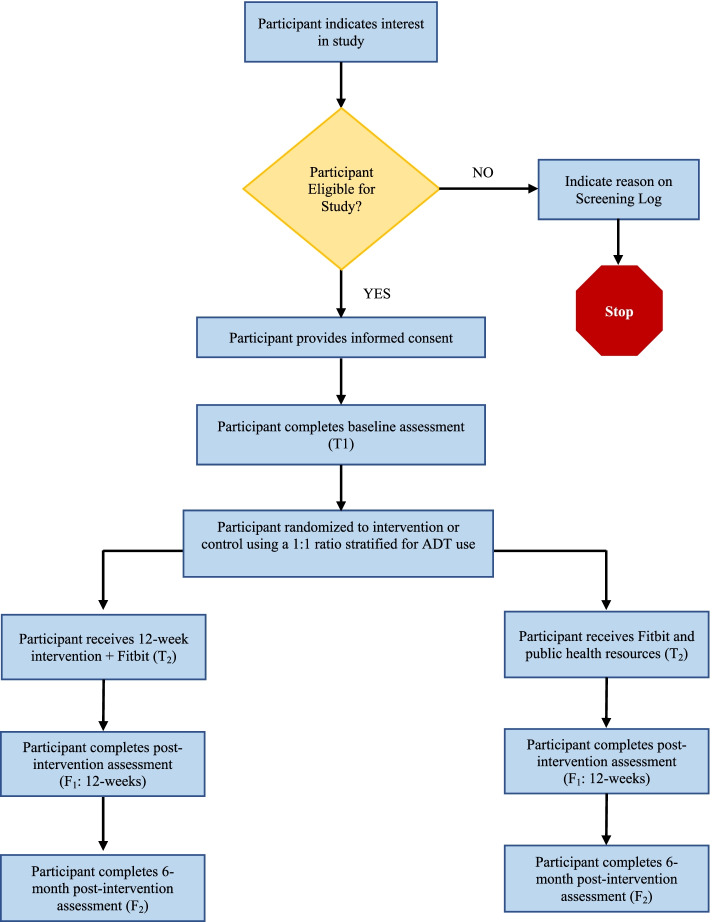
Participant flow through the study

The intervention is a two-armed, parallel groups randomized controlled trial. Following baseline assessments (T_1_), participants will be randomized to one of two groups: 1) an intervention group in which they receive a FitBit, SB workbook, and behavioral counselling or 2) a control group in which they receive a Fitbit and publicly available health education resources (Fitbit-only control). Follow up assessments will take place immediately after the 12-week intervention period (i.e., post-intervention; F_1_) and 9 months from baseline assessments (i.e., 6-month post-intervention; F_2_). The participant flow through the study is summarized in Fig. 1.

### Recruitment and Procedures

#### Participant Eligibility

PCS are eligible to participate if they are: 1) ≥18 years of age; 2) have been diagnosed with localized or asymptomatic metastatic primary prostate cancer; 3) are not currently undergoing radiation or chemotherapy; 4) self-report >8 hours of SB/day during waking hours; 5) are inactive (self-report <150 minutes of MVPA per week); 6) in the contemplation or preparation stage for motivational readiness to change as determined by the Stages of Change Questionnaire [32]; 7) have access to a smartphone, tablet, or computer with a webcam and reliable internet access; 8) received physician clearance to participate if indicated by the Physical Activity Readiness Questionnaire (PAR-Q+); 9) currently reside in Canada and plan to stay in Canada for the next 12 months; and 10) are proficient in English. PCS will be ineligible if they: 1) have a medical condition that prohibits walking (e.g., severe hip or knee arthritis); 2) have been diagnosed with another primary or recurrent invasive cancer (i.e., other than non-melanoma skin cancer); and 3) use a gait aid device.

#### Participant Recruitment

Participants will be recruited from Toronto and Vancouver sites through community partnerships and connections with the genitourinary clinics at the Princess Margaret Cancer Centre in Toronto and the Survivorship and Primary Care Program at BC Cancer. Clinicians will be informed of the study through presentations by the investigative team at rounds, and potential participants will be introduced to the study by their oncologist or clinic nurse. Participants will also be recruited through advertisements in local community cancer organizations, on social media, as well as recruitment e-mails sent out through relevant listservs.

#### Screening Procedure and Informed Consent

Study personnel will complete initial screening of the PCS by phone to ensure they meet the eligibility criteria. Certified exercise physiologists (e.g., American College of Sports Medicine [ACSM], Canadian Society of Exercise Physiologists [CSEP]) who are also part of the study team will provide clearance for eligible participants or obtain physician clearance if required. Written consent will be obtained by all participants in the study.

#### Randomization

Randomization will be performed by study personnel using a stratified randomization scheme via REDCap. The sequence will be random, permutated blocks of varying sizes, stratified by ADT use (i.e., ADT vs. no ADT). PCS will be assigned in a 1:1 ratio to either the intervention or control group. Randomization codes will be kept in a digital file that is separate from other study records. Only the study co-ordinators and investigators will have access to the randomization codes.

#### Blinding

Study personnel and participants will be blind to group allocation during baseline as randomization will take place after completion of all baseline measures. Subsequent data collection appointments will be completed by study personnel that are blind to the group allocation. Due to the nature of the study, it is not possible to blind the participants to their group allocation.

#### Sample Size

A sample size calculation was performed in G*power based on a multivariate analysis of variance with two intervention levels (i.e., intervention, control) and two dependent variables (i.e., SB and PA). Based on our pilot trial with PCS, we assumed a medium effect size of Cohen’s *d* = 0.50 at the end of the intervention. Based on an alpha of 0.05 and power of 0.80, a total sample size of 100 is required. To account for the expected 20% attrition, a sample of 120 PCS (intervention *n* = 60, control *n* = 60) will be recruited.

#### Study Personnel

Study personnel will consist of graduate students and post-doctoral fellows who are certified physiologists/trainers with ACSM or CSEP, with knowledge in behavioral theory, motivational interviewing, health coaching, and exercise oncology. Each participant in the intervention group will be assigned a movement specialist to lead all behavioral counselling sessions. All personnel will be trained by the Principal Investigator to ensure quality control and fidelity (e.g., manual of operations, provider training [3 × 6 hr sessions], intervention delivery checklists) using the treatment fidelity guidelines for health behavior research recommended by the National Institutes of Health (NIH) Behavior Change Consortium [33].

#### Study Conditions

##### Intervention Condition – Fitbit +Behavioral Counselling

The intervention aims to increase daily step counts by ≥3000 steps per day from baseline. The intervention is comprised of five phases which will be delivered through six behavioral counselling sessions (four individual sessions, two group-based webinars). Participants will be provided with a SB workbook and Fitbit Inspire 2 (Fitbit LLC; San Francisco, CA) as an intervention tool to complement the behavioral counselling sessions. The Fitbit Inspire 2 displays steps, distance, calories, sleep/rest time, and provides reminders to move. The Fitbit app syncs wirelessly to computers and smart devices. PCS will be encouraged to wear the device continuously throughout the 12 weeks and to access their SB and activity data at least weekly via the application.

The behavioral counselling sessions are grounded in the M-PAC framework [26] and will take place bi-weekly for 30-60 minutes per session (Table 1). Following randomization, PCS will attend an orientation session with study personnel to introduce them to the Zoom environment and Fitbit. PCS will be asked to self-monitor their daily steps through the Fitbit app and an activity log throughout the 12-weeks. Each of the one-on-one behavioral counselling sessions and group webinars will include activities from the SB workbook to support the behavioral counselling discussions. During the first two weeks (Phase 0), participants will engage in self-monitoring to obtain a baseline step count, which will provide opportunities for tailored goal setting throughout the subsequent phases. Phase I (weeks 3-4) will include a group webinar focusing on reflective processes (i.e., perceived capability and opportunity, instrumental attitudes, and affective judgments) and information about the benefits of reducing SB. PCS will be encouraged to replace SB with LPA by increasing their daily steps by 1000 steps/day above their baseline levels. Phase II (weeks 5-6) and Phase III (weeks 7-8) will focus on behavioral regulation (Phase II: goal setting and action planning; Phase III: coping planning and social support) through one-on-one sessions during each phase (six sessions in total). PCS will be encouraged to increase their daily step counts by 2000 steps/day and 3000 steps/day above their baseline level during Phase II and Phase III, respectively. Phase IV (weeks 9-10) will focus on reflexive processes (i.e., self-regulation consolidation, habit) through a group webinar and a one-on-one session. Phase V (weeks 11-12) will include a final one-on-one booster session to address individual challenges or revisit previous behavioral support topics as needed. Participants will be encouraged to maintain their new daily step count (i.e., 3000 steps/day above baseline) throughout Phases IV and V (see Table 1).

**Table 1:** Delivery of the intervention and behavioral counselling components

Phase (Weeks)	Description				M-PAC Framework [26] Construct	Description of Behavioral Strategies
	Session Type		Focus	Movement Goal^**a**^		
	1-on-1	Group				
Phase 0 (Weeks 1-2)	N/A	N/A	Self-monitoring typical daily sitting time and step counts	No change in activity levels	N/A	N/A
Phase I (Weeks 3-4)		Week 3	Increasing low intensity, incidental movement	↑1000 steps/day	Reflective Processes • Perceived capability • Perceived opportunity • Instrumental attitudes • Affective judgments	• Benefits of reducing sitting time and increasing movement for health and clinical outcomes • How to make activity enjoyable
Phase II (Weeks 5-6)	Week 5		Continuing to increase daily steps and interrupt sitting time	↑2000 steps/day	Behavioral Regulations • Goal setting • Action planning	• Setting challenging, yet achievable goals • Creating plans to increase step counts
Phase III (Weeks 7-8)	Week 7		Increasing activity to longer sessions of activity	↑3000 steps/day	Behavioral Regulations • Coping planning • Social support	• Strategies to overcome barriers to daily movement • How to obtain social support from others
Phase IV (Weeks 9-10)	Week 10	Week 9	Consolidation: combining self-regulatory strategies learned in previous phases	Maintain 3000 steps/day	Reflexive Processes • Self-regulation consolidation • Habit	• Tagging movement behaviors to existing schedule • Utilizing environmental cues • Developing rewards
Phase V (Weeks 11-12)	Week 11		Maintenance: combining self-regulatory strategies learned in previous phases	Maintain 3000 steps/day	Booster Session	• Individual challenges and barriers to setting goals • Revisit previous topics as needed

^a^All goals indicate a change from the participant’s baseline steps identified in Phase 0

#### Active Comparison Condition: Fitbit-only Control

Participants will receive a Fitbit Inspire 2, the Canadian 24-hour Movement Guidelines [34], and a list of free public health resources about maintaining a healthy lifestyle (e.g., Canada’s ParticipACTION app). Following a brief orientation session on how to use the Fitbit and overview of the provided public health resources, the Fitbit-only comparator condition will not receive additional behavioral support from the movement specialists for the intervention period.

### Data Collection

Data will be collected at baseline (T_1_), post-intervention (F_1_), and 6 months post-intervention (F_2_). Participants will complete an electronic questionnaire on REDCap (20 minutes) and will be mailed an activPAL inclinometer. PCS will meet with a movement specialist blinded to group allocation through videoconferencing (i.e., Zoom) to ensure proper fitting of the activPAL, and to complete the physical function test (30 minutes). Study personnel completing data collection will be blinded to group allocation. A summary of outcomes and data collection time points is shown in Table 2. Participants will receive a $60 honoraria for study completion ($20 for each completed assessment; T_1_, F_1_, F_2_).

**Table 2:** Summary of outcome measures

Item	Instrument	Study Period				
		Enrollment	Baseline Assessment/ Allocation	Post-Allocation 12-week Intervention	Post-Intervention	6 months Post- Intervention
		T_0_	T_1_	T_2_	F_1_	F_2_
Eligibility Screen	N/A	X				
Informed Consent	N/A	X				
Allocation	N/A		X			
1. **Primary Outcomes**						
Objectively-measured Sedentary Behavior	ActivPAL		X		X	X
2. **Secondary Outcomes**						
Self-reported Domain-specific Sedentary Behavior	LASA Sedentary Behavior Questionnaire		X		X	X
Objectively-measured Physical Activity	ActivPAL		X		X	X
Self-reported Physical Activity	GLTEQ		X		X	X
Motivational Processes	Standard measures from the M-PAC (15 items)		X		X	X
Mental Health	HADS		X		X	X
Quality of Life	FACT-General, FACT-Fatigue, SF-12		X		X	X
Health and Disability	WHODAS 2.0		X		X	X
Physical Function	30-second chair stand		X		X	X
Internet & Technology Use	eHEALS		X			
Patient Satisfaction	30 items				X	
3. **Covariates**						
Sociodemographics	6 items		X			
Medical History	10 items		X			
**Group Allocation**						
Intervention				X		
Fitbit-Only Control				X		

*Abbreviations*: *LASA* Longitudinal Aging Study Amsterdam, *GLTEQ* Godin Leisure Time Exercise Questionnaire, *M-PAC* Multi-Process Action Control, *HADS* Hospital Anxiety and Depression Scale, *FACT-General* Functional Assessment of Cancer Therapy – General, *FACT-Fatigue* Functional Assessment of Cancer Therapy –Fatigue, *SF-12* Short Form-12, *WHODAS 2.0* World Health Organization Disability Assessment Schedule 2.0, *eHEALS* eHealth Literacy Scale

#### Primary Outcome

##### Sedentary Behavior

Objectively-measured SB will be assessed with an activPAL inclinometer (PAL Technologies, Ltd; Glasgow, UK), which quantifies free-living SB and ambulatory activities. The inclinometer is a small, lightweight device secured to the anterior midline of the right thigh using Tegaderm dressing (3M Medical, USA). It provides time spent in sitting, lying, standing and stepping, as well as estimates energy expenditure expressed as metabolic equivalents (METs) [35]. It has been shown to be a valid and reliable measure compared to direct observation (R^2^ = 0.94) [36], and these devices have been used in cancer populations [11, 37]. Participants will be mailed the inclinometer to wear for seven consecutive days. A wear log will be used to record sleep and wake times, daytime naps, and periods of device removal.

Self-reported SB will be assessed with the Longitudinal Aging Study Amsterdam (LASA) Sedentary Behavior Questionnaire [38]. The LASA measures self-reported sedentary time in ten sitting behaviors on an average weekday and weekend day including occupational, transportation, and leisure-time sitting behaviors (hours and minutes). The questionnaire has a test-retest reliability of 0.71 (95% CI 0.57-0.81) and has been used in older adults [38–41].

### Secondary Outcomes

#### Physical Activity

Self-reported leisure-time PA will be assessed using the Godin Leisure-Time Exercise Questionnaire (GLTEQ) [42, 43]. Participants will report the number of times per week spent in light, moderate, and vigorous leisure-time PA, which will be used to calculate a total leisure activity score. The GLTEQ has been validated and frequently used to assess PA in cancer populations [42, 43]. The GLTEQ will be modified to remove the stipulation to only consider activity completed in bouts of 10 minutes in line with the current PA guidelines for cancer survivors [9]. The GLETQ has good internal consistency with reliability coefficients of 0.83 and 0.85 [42]. Objectively-measured PA will be assessed with the activPALs. A cadence <100 steps/minute will be considered LPA [44, 45].

#### Motivational Processes

Standard M-PAC questionnaires will be modified to assess participants’ reflective (i.e., instrumental and affective attitudes, perceived capability and opportunity), regulatory (e.g., self-monitoring, goal setting, action and coping planning), and reflexive processes (e.g., habit) related to reducing SB. A 7-point Likert scale (strongly disagree to strongly agree) will be used to assess the extent to which participants believe replacing SB with LPA will be exciting/enjoyable/pleasant and wise/beneficial/pleasant to assess *affective* and *instrumental attitudes*, respectively [46]. *Perceived capability* and *opportunity* will be assessed through six statements regarding confidence and control over engaging in LPA to replace SB (e.g., perceived capability: “I possess the skills to replace sedentary time with light PA regularly over the next 12 weeks if I wanted to”; perceived opportunity: “If I really wanted to replace sedentary time with light PA regularly over the next 12 weeks, I would have the chance to do so”) [47, 48]. *Regulatory* and *reflexive* processes will be rated on a 5-point Likert scale (strongly disagree to strongly agree) [49–51]. Example statements include: “I kept track of my daily light PA and sedentary time in a diary or log over the last month” (self-monitoring); “I made plans regarding what to do if something interfered with my plan to replace sedentary time with light PA last month” (coping planning); “Reducing sedentary time to be more active is something I do automatically” (habit). *Intentions* to replace light PA will be assessed using the following prompt: “I intend to engage in regular light PA _________ times per week/weekday/weekend day during the next 12 weeks.”

#### Quality of Life

Disease-specific QoL will be assessed with the Functional Assessment of Cancer Therapy (FACT) – General which consists of the physical well-being (PWB), functional well-being (FWB), emotional well-being (EWB), and social well-being (SWB) subscales [52]. The FACT-Fatigue scale includes the 27 items from the FACT – General plus the 13-item fatigue subscale [52, 53]. On all scales, higher scores indicate better QoL and fewer symptoms. FACT-General and FACT-Fatigue both have good test-retest reliability with correlation coefficients of 0.92 and 0.87, respectively [52, 54].

General health-related QoL will be measured using the Short Form-12 (SF-12). The SF-12 is a 12-item, shortened version of the SF-36 assessing physical functioning, bodily pain, role functioning, emotional well-being, social functioning, energy/fatigue, and health perceptions [55, 56]. It also includes a single item that provides an indication of perceived change in health. The SF-12 has good test-retest reliability correlations of 0.89 and 0.76 for the physical and mental component score, respectively [55]. Additionally, scores from the SF-12 are highly correlated with scores from the complete SF-36 questionnaire [56]. The SF-12 has been previously used in cancer populations [57, 58].

#### Health and Disability

Disability will be assessed using the self-administered 12-item World Health Organization Disability Assessment Schedule 2.0 (WHODAS 2.0) [59]. This questionnaire is used to assess functioning in six major life domains (e.g., cognition, mobility, self-care, getting along, life activities, and participation) [59]. A simple scoring method will be used to sum the 12 items to create a Global Disability total score to describe any functional limitations. The scores for each item ranges from 1 (none) to 5 (extreme) [60]. WHODAS 2.0 has good test-retest reliability with and intra-class correlation coefficient of 0.98 [61].

#### Mental Health

Anxiety and depression will be assessed with the 14-item Hospital Anxiety and Depression Scale (HADS) [62]. The HADS provides anxiety and depression subscale scores and a total score. Each item is rated on a 4-point Likert scale (0 = “not at all”, 3 = “yes, definitely”), and higher scores are indicative of higher distress. The HADS scale has good reliability with intra-class coefficients of 0.92 and 0.88 for the anxiety and depression subscales, respectively [63].

#### Physical Function

Lower body strength will be assessed through the 30-second chair stand from the Senior Fitness Test [64]. This test will be adapted for remote delivery and self-administered by the participant and supervised by study personnel over Zoom [65]. The 30-second chair stand measures the number of full chair stands (seated to a full stand) that can be completed in 30 seconds. Participants will be asked to align a sturdy, non-rolling chair perpendicular to their computer so study personnel can see the full range of movement. Study personnel will start a stopwatch for 30 seconds and count the appropriate number of repetitions that the participant completes. A higher score indicates better lower body strength.

#### Patient Satisfaction

A patient satisfaction survey will be administered post-intervention to assess the satisfaction with the intervention by using researcher-generated, closed-ended questions: “Did the movement specialist effectively demonstrate constructive feedback throughout the program?” “The program workbook was informative and easy to follow?” The survey will assess the burden of the program and the participant’s feedback regarding the SB workbook, counseling sessions, and overall study experience. Likert scales ranging from 1 (not at all) to 7 (very much) will be used. This researcher-generated questionnaire is similar to measures employed in our prior work in PCS [30].

#### Demographic, Clinical, and Technology Literacy Information

The demographic variables include age, gender, marital status, highest level of education, current employment status, ethnicity, PA history, and height and weight to calculate body mass index (BMI). The medical variables include time since diagnosis, disease stage, current/prior treatments, previous recurrence, and current disease status, which have been used previously in studies with cancer survivors [20, 21, 66]. Technology literacy will be assessed using a modified version of the eHEALS ehealth literacy scale and 10 additional questions about internet and social media usage [67]. The eHEALS is an 8-item measure of eHealth literacy designed to measure combined knowledge, comfort, and perceived skills at finding, evaluating, and applying electronic health information to health problems [67]. The eHEALS has moderate stability with a test-retest correlations between 0.49 to 0.68 [67]. The eHEALS will be adapted to assess participants’ confidence in using and finding online information related to SB and PA specifically. The questions will assess general patterns of internet and social media use, including methods used to access the internet and social media, frequency of use, devices owned, and types of social media accounts owned [68].

##### Statistical Analyses

Linear mixed models will be used to model the primary outcome (i.e., SB) and secondary outcomes (i.e., LPA, motivational outcomes from M-PAC, QoL) at the three time points. Fixed effects included in the models will be time (i.e., baseline, 3-month, 6-month post-intervention), group (i.e., intervention, control), and the interaction. A random intercept will be included to account for variance between and within participants. All models will be estimated while adjusting for covariates (e.g., baseline SB, age, treatment type). Sensitivity analyses will be performed to test for informative dropouts.

The same analytic approach will assess subgroup differences of participants which will include several standard demographic (e.g., age, gender), fitness (e.g., baseline PA) and clinical variables (e.g., time since treatment, disease stage) by modeling the changes over time from post-intervention (F_1_: 3-months after baselines) to 6-month follow-up (F_2_: 9-months after baseline). An intention to treat analysis will be conducted based on all available data from PCS randomized regardless of non-adherence during the intervention. Participants with missing data will be included under the missing-at-random assumption of the mixed-model analysis.

##### Monitoring

A data audit will take place at least once a month to ensure all forms are completed accurately. Depending on the recruitment rate, the data audit may take place more frequently. In terms of safety monitoring, all adverse events will be classified as either an adverse (AE) or a serious adverse event (SAE). These events may occur during screening and baseline data collection, or they may occur during the administration of the intervention. An adverse event may or may not be related to the data collection or intervention procedures. When an adverse event is identified, study personnel identifying the adverse event will report it to the study co-ordinator and the principal investigator. The study co-ordinator will then contact the participant and an electronic Adverse Event Report Form will be submitted to the research ethics board will be completed within 7 days of the event and within 48 hours for serious adverse events. Decisions to discontinue or modify the intervention for a participant will be on a case-by-case basis by the research team.

## Discussion

Developing and testing distance-based interventions that replace SB with PA are warranted for several reasons. First, supervised PA has a positive impact on clinical outcomes such as QoL and reducing treatment-related toxicities in PCS [7, 69, 70]. However, access to such interventions may be limited by cost, geographic reach, availability of qualified PA professionals, and safe facilities given transmission concerns from the recent COVID-19 pandemic. Even when supervised PA programs are offered to cancer survivors at low cost, uptake and adherence may be low at intervention completion [14]. Second, health behaviors such as PA and SB are important in the self-management of cancer. Men with prostate cancer indicate that they are more likely to seek information on the internet [71]. Therefore, there is a need for evidence-based interventions that are easy to access, low-cost and scalable. This is particularly salient during the pandemic given that being physically active in public spaces presents risks for transmission in vulnerable populations such as cancer survivors [72]. Adapting usual methods of lifestyle program delivery to home-based programs for general well-being, mitigating treatment toxicities, and improving clinical outcomes are needed. One such intervention is digital health behaviour change. Digital interventions use technologies such as text messaging, e-mail, mobile applications, videoconferencing, social media, websites, and online patient portals. These technologies increase access to information, connecting patients with health services as an approach to remote delivery of behaviour change interventions. Such technologies have been used successfully in promoting PA participation and dietary behaviours in cancer survivors [72].

Given that cancer survivors may face several barriers to engaging in PA programs, there is a need to develop and assess the efficacy of interventions that employ distance-based approaches (e.g., characterized by limited face-to-face contact and/or supervision) [73]. Groen et al. [73] conducted a systematic review examining distance-based PA behavior change interventions for cancer survivors where the results show that intervention effects on PA were small and new approaches were needed. The current study will build on our successful pilot study by using a more robust clinical design and focusing on maintenance of behavior change in two important ways. A series of behavioral counselling sessions based on the M-PAC will be adapted throughout the trial that move beyond intention formation to focus on maintenance. This is noteworthy as theory-based interventions result in meaningful changes in behavior compared to atheoretical approaches in cancer survivors [74], including PCS [75]. Finally, our prior work was a single-group feasibility study, while this current study will examine the effects of the intervention on SB in a well-powered RCT. Overall, the current intervention will move from a feasibility to an efficacy trial, focus on maintenance grounded in theory (e.g., M-PAC), include sustainable wearables (e.g., Fitbit) that could be integrated within typical delivery models of cancer care.

Cancer survivorship is now recognized as an essential cancer care component, and greater efforts on maintaining QoL is needed. Cancer Care Ontario published specific follow-up care guidelines for PCS [76]. These guidelines aim to promote comprehensive follow-up care, optimal health, and QoL in PCS, which includes health promotion (i.e., PA) and management of physical and psychosocial side effects. Therefore, decreasing SED and increasing PA may be an effective health promotion strategy for PCS. The current intervention has high potential for broad reach and impact on both PCS and their support networks, as it can ultimately be delivered safely through internet- and mobile-based applications. With internet usage growing fastest among older Canadians [77], our study will create a unique distance-based platform that could be scaled for use by clinical and community-based organizations as a low-cost, supportive care tool to improve QoL for all cancer survivors across Canada.

## Data Availability

The datasets used and/or analysed during the current study are available from the corresponding author on reasonable request.

## Abbreviations

ACSM: American College of Sports Medicine
ADT: Androgen Deprivation Therapy
BMI: Body Mass Index
CSEP: Canadian Society of Exercise Physiologists
EWB: Emotional Well-being
eHEALS: eHealth Literacy Scale
FACT: Functional Assessment of Cancer Therapy
FWB: Functional Well-being
GLETQ: Godin Leisure-time Exercise Questionnaire
HADS: Hospital Anxiety and Depression Scale
LASA: Longitudinal Aging Study Amsterdam
LPA: Light Physical Activity
MVPA: Moderate-to-vigorous physical activity
M-PAC: Multi-Process Actional Control Approach
PA: Physical Activity
PAR-Q+: Physical Activity Readiness Questionnaire Plus
PASSS: Physical Activity and Social Support Scale
PCS: Prostate Cancer Survivors
PSQI: Pittsburgh Sleep Quality Index
PWB: Physical Well-being
RA: Research Assistant
REDCap: Research Electronic Data Capture
SB: Sedentary Behavior
SF-12: Short-form-12
SWB: Social Well-being
QoL: Quality of Life
WHODAS 2.0: World Health Organization Disability Assessment Schedule 2.0
